# Pharmacokinetics of Rifabutin in Japanese HIV-Infected Patients with or without Antiretroviral Therapy

**DOI:** 10.1371/journal.pone.0070611

**Published:** 2013-08-05

**Authors:** Junko Tanuma, Kazumi Sano, Katsuji Teruya, Koji Watanabe, Takahiro Aoki, Haruhito Honda, Hirohisa Yazaki, Kunihisa Tsukada, Hiroyuki Gatanaga, Yoshimi Kikuchi, Shinichi Oka

**Affiliations:** 1 AIDS Clinical Center, National Center for Global Health and Medicine, Tokyo, Japan; 2 Department of Drug Metabolism and Disposition, Meiji Pharmaceutical University, Tokyo, Japan; Fundacion Huesped, Argentina

## Abstract

**Objective:**

Based on drug-drug interaction, dose reduction of rifabutin is recommended when co-administered with HIV protease inhibitors for human immunodeficiency virus (HIV)-associated mycobacterial infection. The aim of this study was to compare the pharmacokinetics of rifabutin administered at 300 mg/day alone to that at 150 mg every other day combined with lopinavir-ritonavir in Japanese patients with HIV/mycobacterium co-infection.

**Methods:**

Plasma concentrations of rifabutin and its biologically active metabolite, 25-*O*-desacetyl rifabutin were measured in 16 cases with HIV-mycobacterial coinfection. Nine were treated with 300 mg/day rifabutin and 7 with 150 mg rifabutin every other day combined with lopinavir-ritonavir antiretroviral therapy (ART). Samples were collected at a median of 15 days (range, 5–63) of rifabutin use.

**Results:**

The mean C_max_ and AUC_0–24_ of rifabutin in patients on rifabutin 150 mg every other day were 36% and 26% lower than on 300 mg/day rifabutin, while the mean C_max_ and AUC_0–24_ of 25–*O*-desacetyl rifabutin were 186% and 152% higher, respectively. The plasma concentrations of rifabutin plus its metabolite were similar between the groups within the first 24 hours, but it remained low during subsequent 24 to 48 hours under rifabutin 150 mg alternate day dosing.

**Conclusion:**

Rifabutin dose of 150 mg every other day combined with lopinavir-ritonavir seems to be associated with lower exposure to rifabutin and its metabolite compared with rifabutin 300 mg/day alone in Japanese patients. Further studies are needed to establish the optimal rifabutin dose during ART. The results highlight the importance of monitoring rifabutin plasma concentration during ART.

**Trial registration:**

UMIN-CTR (https://upload.umin.ac.jp/cgi-open-bin/ctr/ctr.cgi?function=search&action=input&language=E) UMIN000001102

## Introduction

Rifabutin is commonly used for human immunodeficiency virus (HIV)-associated mycobacterial infections, especially during combination antiretroviral therapy (cART) containing HIV protease inhibitors (PIs), since it is less likely to induce hepatic microsomal enzymes than rifampicin [1]–[4]. Conversely, rifabutin and its active metabolite, 25–*O*-desacetyl rifabutin, are substrates of CYP 3A4 and concomitant use of PIs can elevate blood concentrations of rifabutin and 25–*O*-desacetyl rifabutin [3]–[8]. Such rise can increase the risk of side effects such as anterior uveitis [2], [9]–[12]. Thus, a lower dose of rifabutin has been recommended in patients treated with PIs.

The previously recommended dose of rifabutin in combination with ritonavir-boosted PI (PI/r) [13] of 150 mg every other day, was associated with low rifabutin plasma concentrations and increases rate of acquired rifamycin resistance [14]–[17]. Furthermore, the Tuberculosis Trials Consortium (TBTC)/US Public Health Service Study 23 [14] suggested that AUC0–24 of 4.5 μg/mL is the cutoff value for risk of emergence of resistance to rifamycin. On the other hand, the combination of rifabutin at 150 mg thrice weekly with atazanavir-ritonavir provides exposure to rifabutin comparable to that of rifabutin 300 mg alone [11]. Thus, although 150 mg/day is the current recommended dose for rifabutin during PI/r-based cART [4], the optimal dose of rifabutin when used with a PI/r regimen remains to be established.

Ethnic differences, including body weight, renal clearance and various genetic factors like single nucleotide polymorphism (SNP), haplotype or DNA methylation [18], [19], may alter the dose required to achieve a particular concentration of the drug in the circulation. Thus, pharmacokinetic studies involving different ethnic groups are needed to determine the recommended dose that take such factors into account. To our knowledge, there are no such pharmacokinetic studies for rifabutin use in Asians, who are characterized by lower body weight compared with other ethnic groups. The present study was conducted to evaluate the pharmacokinetics of rifabutin in Japanese patients with HIV-1-related mycobacterial infection when used alone at 300 mg/day without cART and at 150 mg every other day when used in combination with lopinavir/ritonavir.

## Methods

### Ethics Statement

The study protocol was approved by the Ethics Committee of the National Center for Global Health and Medicine (NCGM-H20–580: approved on 7th February 2008). All participants provided their written informed consent before enrollment as indicated in the protocol.

The protocol for this study and supporting CONSORT checklist are available as supporting information; see File S1 for English translation of the protocol and File S2 for the Japanese original protocol and File S3 for CONSORT checklist.

### Study design

Consecutive patients with HIV-1-related mycobacterial infection who received rifabutin-containing therapy at the National Center for Global Health and Medicine, Tokyo, Japan, between February 2008 and March 2009, were eligible for the study. After their written informed consent was provided, clinical history, physical examinations and laboratory tests (e.g., blood chemistry and complete blood cell count) were carried out within one week prior to the pharmacokinetic study. Patients were excluded if they were over 20 years of age or if they had abnormal liver function tests [aspartate aminotransferase (AST), alanine aminotransferase (ALT) or total bilirubin (>3 times the upper limit of normal: ULN)], or severe renal dysfunction (creatinine clearance <30 ml/min), and in the case of female patients if they were pregnant or breastfeeding. Rifabutin was administered while fasting at 300 mg/day and the dose was adjusted when used with cART as recommended by the treatment guideline at the time of the study [13]. Medications administered concomitantly or within 2 weeks before the first study day were recorded. To evaluate the impact of rifabutin plasma concentration on treatment efficacy and adverse events, participants were followed up for at least 2 years after stopping rifabutin. Any side effect noted during rifabutin use or within four weeks after stopping rifabutin, its association with rifabutin was assessed.

### Pharmacokinetic assays

Pharmacokinetic sampling commenced after 5 days of rifabutin-containing anti-mycobacterial therapy without (Group I) or with (Group II) cART. Sequential enrollment of a patient into both groups was accepted. Blood samples were collected just before rifabutin administration and then 0.5, 1, 2, 4, 6, 8 and 24 hours afterward. Patients of Group II treated with 150 mg of rifabutin every other day underwent additional sampling at 48 hours. The plasma concentrations of rifabutin and its major metabolite, 25–O-desacetyl rifabutin [20]–[23] were determined simultaneously by validated high-pressure liquid chromatography (HPLC). Blood samples were taken in heparin-containing tubes, placed on ice and centrifuged at 3000×g for 10 min. Then, the obtained plasma was deproteinized by using three times volume of methanol and centrifuged 15,000×g for 5 min, and the supernatant was used for assay. The HPLC standard for rifabutin and 25-O-desacetyl rifabutin were kindly provided by Pfizer Co. (Pfizer, Inc., NY). The HPLC system consisted of Agilent 1100 series (Agilent Technologies, Santa Clara, CA). Isocratic elution was performed using the Inertsil ODS-3 column (5 µm, 4.6 mm I.D. ×150 mm; GL Sciences Inc, Tokyo, Japan) with a guard column (5 µm, 4.6 mm I.D. ×10 mm; GL Sciences Inc). The UV detection wavelength was 280 nm. The mobile phase consisted of 9 mM phosphate buffer (pH 6.8)-acetonitrile (30∶70, v/v). The flow-rate was set at 1.0 ml/min and all separations were performed at 30°C in column oven.

### Statistical and pharmacokinetic analyses

The area under the curve (AUC) was calculated using non-compartmental techniques (WinNonlin, ver. 5, Pharsight Corp., Mountain View, CA) based on the obtained values (AUC 0–24 h for all, AUC 0–48 h for Group II). The maximum plasma concentration (C_max_) and time of C_max_ (T_max_) were determined directly from the data.

Statistical analyses were performed using SPSS software package for Windows, version 17.0J (SPSS Japan Inc, Tokyo). Differences between groups were determined by using the Fisher's exact test for categorical data and the Mann Whitney's test for continuous variables. For all statistical analyses, differences were considered significant if the p value was less than 0.05.

## Results

### Patient characteristics

**Figure 1:**
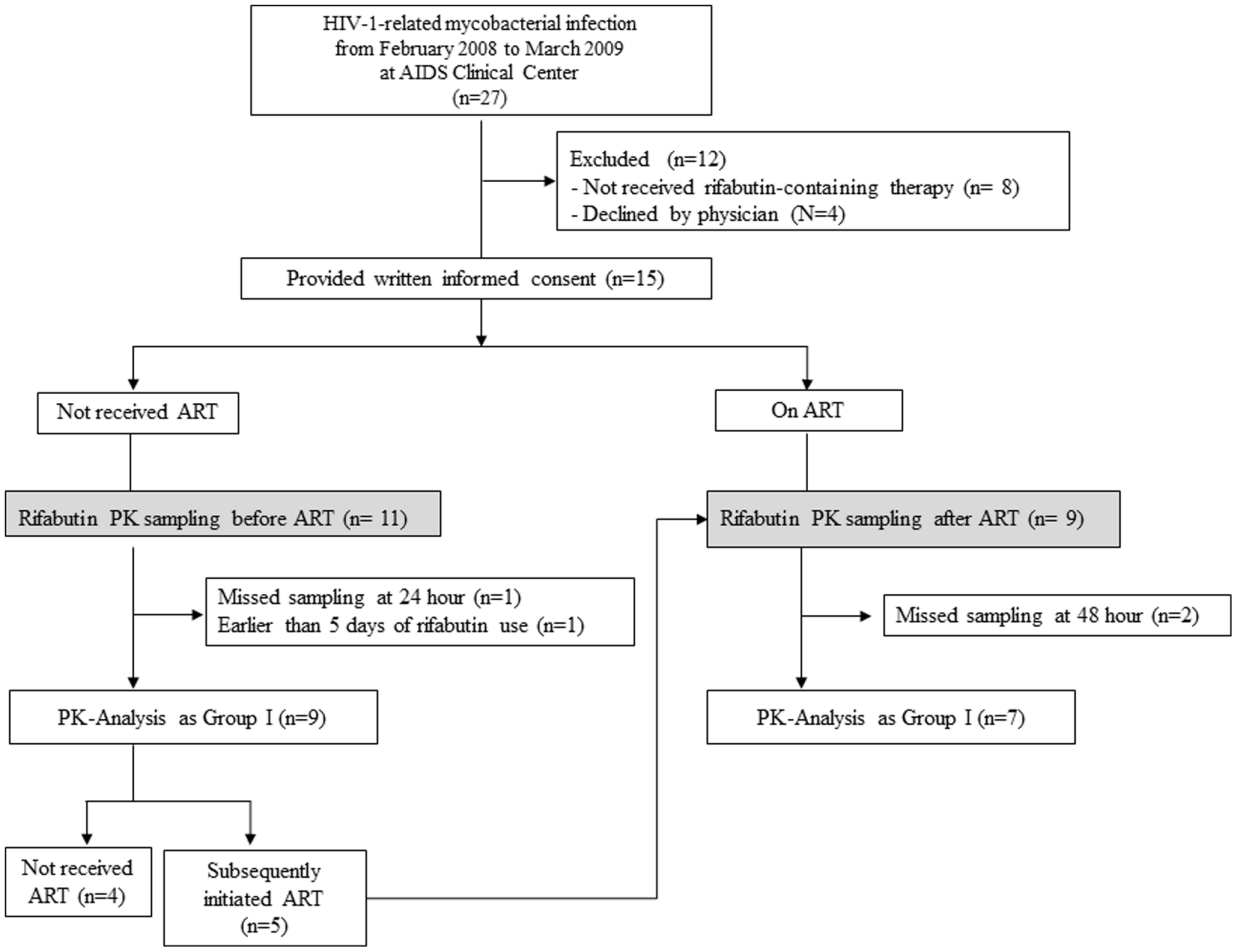
Flow chart of participants through the study. PK, pharmacokinetic; ART, antiretroviral therapy.

A total of 15 patients were enrolled in the study and 5 of 15 participated in both Group I and II. In total, twenty sampling was done for rifabutin pharmacokinetic analysis; 11 in Group I and 9 in Group II. Data from two sampling in Group I and 2 in Group II were excluded from the analysis because samples at 24-hour were unavailable or sampling was conducted earlier than 5 days of rifabutin use. As a result, data from 9 sampling in Group I and 7 sampling in Group II were used for analysis. The baseline characteristics of the 16 sampling cases are summarized in Table 1. All 7 patients of Group II were being treated with lopinavir/ritonavir as their cART, and thus rifabutin was administered at 150 mg every other day based on the guidelines at the time of the study [13]. Two cases of Group I and 1 of Group II were being treated with clarithromycin (CAM) [20] for systemic mycobacterial infection caused by *M. avium *or *M. intracullulare* (*M. avium* Complex: MAC). Five patients of Group I, in whom ART had been delayed several weeks after anti-mycobacterial therapy to prevent the immune reconstitution inflammatory syndrome (IRIS), were later enrolled in the study as patients of Group II (Figure 1). Accordingly, the median time of rifabutin use was longer in Group II than in Group I. There was no significant difference between the groups with regard to gender, age, body weight, CD4 counts, HIV-RNA load, type of mycobacteria and concomitant use of clarithromycin or fluconazole. All were Japanese and the median body weight was 57.3 kg. All patients completed their anti-mycobacterial treatment with clinical resolution of mycobacterial infections. None of the participants had treatment failure or relapse within more than 3 years of observation. Worsening of intra-abdominal lymphadenitis was observed in one patient with systemic M. avium infection at 8 months after stopping the 2-year rifabutin-containing anti-mycobacterial therapy, which excluded treatment failure or relapse. All patients confirmed complete adherence to anti-mycobacterial therapy and cART.

**Table 1:** Characteristics of study subjects.

	All (n = 16)	Group I (without cART, n = 9)	Group II (with cART, n = 7)	*p* value a
Male sex, n	16	9	7	
Age, median years (range)	36 (23–60)	36 (23–55)	35 (23–60)	0.53
Body weight, median kg (range)	57.3 (44–66)	58.0 (46–64)	56.5 (44–66)	0.98
Mycobacterium, multiple choice, n				
*M. tuberculosis*	13	7	6	1.00
*M. avium*	4	3	1	0.94
*M. kansasii*	1	0	1	0.85
CD4 count, median cells/mm^3^ (range)	63 (2–164)	63 (2–164)	63 (19–135)	0.84
Plasma viral load, median log copies/ml (range)	4.97 (3.43–6.62)	4.98 (4.18–6.62)	4.95 (3.43–5.18)	0.10
AST, median IU/L (range)	29 (16–70)	25 (16–59)	30 (17–51)	0.65
ALT, median IU/L (range)	27 (13–70)	26 (23–70)	29 (19–70)	0.31
Time on rifabutin, median days (range)	15 (5–63)	7 (5–20)	29 (10–63)	0.017
Time on cART, median days (range)	14 (10–29)	–	14 (10–29)	–
Concomitant medications, n				
lopinavir-ritonavir	7	–	7	–
clarithromycin	3	2	1	1.00
fluconazole	1	0	1	0.85

a By Fisher's exact test for categorical data and Mann Whitney's U test for continuous variables.

cART, combination antiretroviral therapy; AST, aspartate aminotransferase; ALT, alanine aminotransferase; IU, international unit.

### Pharmacokinetic parameters of rifabutin and its 25-*O*-desacetyl metabolite

**Figure 2:**
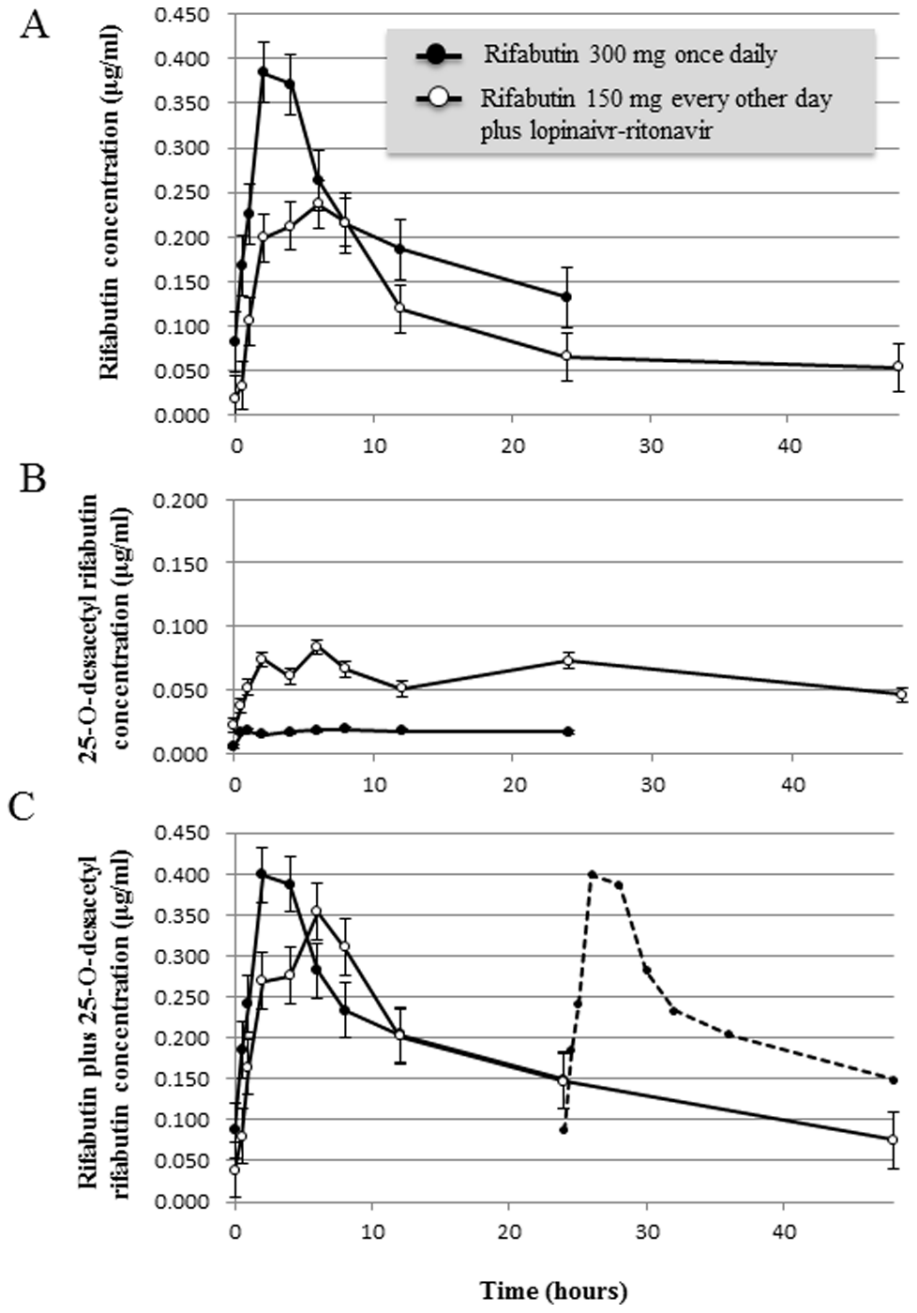
Mean plasma concentrations-versus-time plots of rifabutin (A), 25-*O*-desacetyl rifabutin (B), and rifabutin plus 25-*O*-desacetylrifabutin (C). Nine patients of Group I received 300 mg of rifabutin and 7 patients of Group II received 150 mg of rifabutin every other day with lopinavir/ritonavir-containing antiretroviral therapy. *Solid circles:* Group I, *open circles:* Group II. Data are mean ±1 standard errors. Dotted line in Figure C represents data of Group I during 0–24 hour for reference. RBT, rifabutin; PI/r, ritonavir-boosted protease inhibitor.

The pharmacokinetic parameters of rifabutin and 25-*O*-desacetyl rifabutin are summarized in Table 2 and their mean plasma concentration-time data of 48 hours are illustrated in Figure 2A and 2B. For calculation of AUC_0–48_, the data from 24 to 48 hours in Group I was assumed to be the same as that for 0–24 hours because rifabutin was administered once a day at the same dosage. As shown in Table 2, the mean values of C_max_ and AUC_0–24_ of rifabutin were 36% and 26% lower in Group II than in Group I, while the mean values of C_max_ and AUC_0–24_ of 25-*O*-desacetyl rifabutin were 186% and 152% higher in Group II than in Group I. However, the differences in the above values between the two groups were not statistically different. The low rifabutin concentration and high metabolite concentration in Group II may reflect the induction of rifabutin metabolism due to the longer duration of rifabutin use. Since 25-*O*-desacetyl rifabutin is microbiologically active against mycobacterium, total rifabutin activity might include rifabutin plus this metabolite. Figure 2C illustrates the mean plasma concentration of rifabutin plus the metabolite over time. Patients of Groups I and II had similar plasma concentrations of rifabutin plus the metabolite within the first 24 hours. However, the level of rifabutin plus the metabolite during the subsequent 24–48 hours was considerably lower in Group II than in Group I (dotted line in Figure 2C: Group I during 0–24 hours), whereas the AUC_0–48_ was not statistically different between the groups. Notably, 6 (67%) cases of Group I and 5 (71%) of Group II failed to achieve the AUC_0–24_ value suggested as risk for emergence of rifamycin-resistant *M. tuberculosis* [14] (4.5 μgh/mL). Neither C_max_ nor AUC_0–24_ of rifabutin and 25-*O*-desacetyl rifabutin were associated with age, body weight, body mass index, or CD4 count.

**Table 2:** Pharmacokinetic parameters for rifabutin and 25-*O*-desacetyl rifabutin.

	Group I (without combination antiretroviral therapy, n = 9)				Group II (with combination antiretroviral therapy, n = 7)				*P* value^a^
	Median (range)		Mean (90% CI)		Median (range)		Mean (90% CI)		
Rifabutin									
C_max_ (μg/mL)	0.46	(0.15–0.86)	0.44	(0.39–0.49)	0.28	(0.10–0.44)	0.29	(0.25–0.33)	0.10
AUC_0–24_ (μg h/mL)	2.79	(1.32–15.7)	4.86	(3.83–5.90)	3.00	(1.13–5.43)	3.38	(2.92–3.84)	0.38
AUC_0–48_ (μg h/mL)^b^	5.59	(2.63–31.3)	9.71	(7.62–511.8)	4.21	(1.76–6.90)	4.58	(3.38–5.78)	0.32
T_max_ (h)	2.0	(2.0–4.0)	2.9	(2.6–3.1)	6.0	(2.0–12.0)	4.8	(4.1–5.1)	0.03
25-*O*-desacetyl rifabutin									
C_max_ (μg/mL)	0.00	(0.00–0.30)	0.05	(0.03–0.08)	0.13	(0.05–0.23)	0.14	(0.12–0.16)	0.05
AUC_0–24_ (μg h/mL)	0.00	(0.00–3.69)	0.82	(0.45–1.20)	1.52	(0.44–3.64)	2.07	(1.62–2.52)	0.12
AUC_0–48_ (μg h/mL)^b^	0.00	(0.00–7.38)	1.64	(0.89–2.39)	5.93	(0.44–7.21)	4.32	(3.27–5.38)	0.15
T_max_ (h)	6.0	(2.0–8.0)	5.3	(4.6–6.0)	6.0	(2.0–12.0)	5.7	(4.6–6.9)	0.87
Rifabutin plus 25-*O*-desacetyl rifabutin									
C_max_ (μg/mL)	0.47	(0.15–0.99)	0.49	(0.40–0.52)	0.42	(0.16–0.56)	0.39	(0.34–0.44)	0.54
AUC_0–24_ (μg h/mL)	3.36	(1.32–19.3)	5.49	(4.18–6.76)	6.23	(1.57–7.92)	5.27	(4.48–6.07)	0.93
AUC_0–48_ (μg h/mL)^b^	6.72	(2.63–38.7)	10.9	(8.35–13.5)	6.80	(2.20–14.1)	7.95	(6.40–9.49)	0.46

a By the Mann Whitney's *U* test.

b In Group I, AUC_24–48_ is assumed the same as AUC_0–24_ and AUC_0–48_ is calculated as double of AUC_0–24_ for comparison with Group II.

C_max_, maximum plasma concentration; AUC, area under the curve; T_max_, time of C_max_; CI, confidence interval.

### Rifabutin-associated side effects

Of the 15 participants, three patients developed side effects possibly related to rifabutin during the observational period; two of Group I developed skin rash and the other of Group II developed grade 2 rise in liver enzymes (ALT or AST 2.6–5.0 times of ULN). The skin rash appeared on day 11 of rifabutin-containing regimen in one patient and on day 28 in the other, and was resolved in both patients within several days after withdrawal of rifabutin. The rise in liver enzymes was detected after two months of rifabutin-containing regimen in combination with cART, and improved soon after discontinuation of rifabutin. Notably, the median CD4 counts in the three patients with rifabutin toxicity were significantly lower than in patients without rifabutin toxicity (12 *vs* 76, cells/mm^3^, p = 0.028). However, rifabutin toxicity did not correlate with rifabutin AUC_0–24_, C_max_, or the concurrent use of cART (rifabutin AUC_0–24_: p = 0.37, rifabutin C_max_: p = 0.86, cART use: p = 0.21).

## Discussion

In the present study, a low dose of rifabutin (150 mg every other day), in combination with lopinavir/ritonavir-containing cART, yielded comparable AUC_0–24_ of rifabutin and 25-*O*-desacetyl rifabutin to the commonly used dose of rifabutin of 300 mg/day. The advantage of the low-dose rifabutin included lower exposure to rifabutin and metabolite during the subsequent 24 to 48 hours in Japanese patients with HIV-mycobacteria co-infection. Since many participants started their cART after at least 1 month of anti-mycobacterial therapy in order to avoid deterioration by immune-reconstitution syndrome, the metabolism of rifabutin was induced upon the commencement of cART. This led to lower rifabutin concentration and higher 25-*O*-desacetyl rifabutin concentration in Group II but provided similar concentrations of rifabutin plus its active metabolite. However, on the day without medication, plasma concentrations of rifabutin and its active metabolite were lower in Group II, which were less than the susceptibility breakpoint level for *M. tuberculosis* proposed by others [20]. This suggests increased risk of emergence of rifamycin-resistant *M. tuberculosis* during the day without medication under low-dose rifabutin therapy, and that the currently recommended dosage 150 mg daily with PI/r is reasonable to this population as well. In this regard, Zhang et al. [11] reported that treatment with 150 mg/day rifabutin with atazanavir-ritonavir resulted in high risk of severe neutropenia. Furthermore, their post-hoc simulation showed that rifabutin 150 mg thrice weekly with atazanavir-ritonavir provided a comparable exposure to rifabutin compared with rifabutin 300 mg daily. Considering the risk of rifamycin-resistance and rifabutin toxicity, monitoring of rifabutin plasma concentration should be considered until the optimal rifabutin dosing during PI/r-based cART is fully established.

Although none of the patients showed treatment failure or relapse in this study, the rifabutin AUC0-24 observed in the study was in general close to the low end of the value reported in previous studies [7], [14] and many participants [6 (67%) of Group I and 5 (71%) of Group II] failed to achieve AUC0-24 4.5 μgh/mL, the cutoff value suggested as risk for emergence of rifamycin-resistant M. tuberculosis [14]. One of the reasons for this discordant result might be the limitation of our study of small sample size involving several MAC and M. kansasii infections. Since acquisition of rifamycin-resistance M. tuberculosis was not frequent enough in this study group, it was difficult to evaluate the association with rifabutin pharmacokinetics and emergence of rifamycin-resistance. Other reasons may be the biological characteristics of rifabutin. Rifabutin has long postantibiotic effect against M. tuberculosis and MAC [20], shows extensive distribution in various tissues [21], [22], and readily penetrates cell membranes of leucocytes [21], [22]. These characteristics and their variations among patients can considerably influence the outcome of rifabutin-containing anti-mycobacterial therapy and therefore might be one of the explanations of favorable efficacy despite lower plasma concentrations of rifabutin in our study. Another limitation of this study is that plasma concentration of isoniazid was not measured, although low isoniazid plasma concentration is known to be independently related to treatment failure of HIV/TB co-infection [24]. Additionally, although there was no difference in rifabutin concentration among the patients with or without use of clarithromycin or fluconazole, those drugs can increase the rifabutin AUC and possibly affect the results. Since our study was enrolling patients with heterogeneous backgrounds in the real clinical setting, such as timing of sampling or different combination of anti-mycobacteial drugs, it was difficult to completely eliminate those impacts from the analysis. These conditions should be taken into account in the assessment of treatment outcome and associated factors in this study.

Among 15 study participants, 3 patients developed side effects related to rifabutin therapy, including skin rash and rise in liver enzymes. Notably, their CD4 counts were lower than those who did not show rifabutin toxicity, although rifabutin plasma concentrations and the concurrent use of cART were similar in the two groups. This is the first report implicating low CD4 count as a risk factor for rifabutin-related side effects. However, like other side effects of rifabutin, such as uveitis and leukocytopenia, which have been reported to be related to high-dose rifabutin or high rifabutin plasma concentrations [9]–[12], careful assessment involving larger population samples are needed to evaluate the association between high plasma concentrations of rifabutin and the related skin rash and hepatotoxicity.

In conclusion, in Japanese patients with HIV-mycobacteria co-infection, the plasma concentrations of rifabutin and active metabolite within the first 24 hours of treatment with low-dose rifabutin (150 mg every other day) combined with lopinavir-ritonavir, were similar to those encountered with 300 mg/day rifabutin alone. However, these concentrations decreased on the day without medication. Our findings could help determine the optimal dose of rifabutin during cART. Further studies are needed to establish the optimal dose of rifabutin during cART. Monitoring of rifabutin plasma concentration should be considered in patients with HIV-mycobacteria co-infection.

## Supporting Information

Protocol S1 **Summary in English.** English translation of the protocol Summary.
(DOCX)

Protocol S1 **Protocol and IC form in Japanese.** The full version of the study protocol and the informed consent form in Japanese.
(PDF)

CONSORT **Checklist S2**
(DOC)
