# Supplementary material for: Pharmacokinetics of Rifabutin in Japanese HIV-Infected Patients with or without Antiretroviral Therapy
Source: PLoS One. 2013 Aug 5;8(8):e70611. doi: 10.1371/journal.pone.0070611 (PMC3734254; doi:10.1371/journal.pone.0070611)
Supplement: Protocol S1 — Summary in English. English translation of the protocol Summary. (DOCX) [file pone.0070611.s001.docx]

**Pharmacokinetic Study of rifabutin in Japanese HIV-infected patients with or without antiretroviral therapy**

Study Protocol Ver2.0

Updates

Ver 1.0, 19 December, 2007

Ver 2.0, 23 January, 2008

Primary Investigator:

Junko Tanuma, M.D., AIDS Clinical Center, National Center for Global Health and Medicine

Sponsor:

The Health Labour Sciences Research Grant (#H18-AIDS) from the Ministry of Health, Labour and Welfare, Government of Japan.

**1. BACKGROUNDS AND RATIONALE**

Rifabutin is commonly used for human immunodeficiency virus (HIV)-associated mycobacterial infections, especially during highly active antiretroviral therapy (HAART) containing HIV protease inhibitors (PIs), since it is less likely to induce hepatic microsomal enzymes than rifampicin. Ethnic differences may alter the dose required to achieve a particular concentration of the drug in the circulation, while there are no such pharmacokinetic studies for rifabutin use in Asians.

The present study was conducted to evaluate the pharmacokinetics of rifabutin in Japanese patients with HIV-1-related mycobacterial infection when used alone at 300 mg/day without HAART and at 150 mg every other day when used in combination with protease inhibitors.

**2. OBJECTIVES**

**# Primary objective**

To evaluate the pharmacokinetics of rifabutin in Japanese HIV-infected patients.

**# Secondary objective**

To investigate the impact of protease inhibitors on the pharmacokinetics of rifabutin in Japanese HIV-infected patients.

**3. STUDY DESIGN**

An open-label pharmacokinetic study with two arms

**4. METHODS**

**4.1 Inclusion criteria**

Japanese HIV-infected individuals over 20 years of age who receive rifabutin-containing anti-mycobacterial therapy at National Center for Global Health and Medicine (formerly, International Medical Center of Japan, IMCJ) and provide written informed consent.

**4.2 Exclusion criteria**

- Abnormal liver function tests [aspartate aminotransferase (AST), alanine aminotransferase (ALT) or total bilirubin (>3 times the upper limit of normal: ULN)],

- Renal dysfunction (creatinine clearance <30 ml/min)

- During pregnancy or breastfeeding

**4.3 Study group and sample size**

**Group I** (10 patients): Rifabutin 300 mg/day without HAART

**Group II** (10 patients): Rifabutin 150 mg/day every other day combined with PI containing HAART

Since the average number of HIV-positive mycobacterium cases at NCGM is around ten per year, at least 10 for each study arm is expected if the sequential enrollment of a patient into both groups is achieved in most participants.

**4.4 Study period**

**Enrollment:** from February 2008 and March 2009.

**Follow-up observation:** two years after discontinuation of rifabutin use.

**4.5 Demographic and Clinical data**

Demographic and clinical information will be collected in all participants as followings; age, gender, route of HIV-1 infection, body weight, height, CD4+ T lymphocyte count, HIV-1 plasma viral loads, HBs antigen, anti-HCV antibody, AST, ALT, total bilirubin, serum creatinine, and any adverse events of grade 2 or greater from the commencement of rifabutin use until two years after discontinuation of rifabutine use.

**4.6 Pharmacokinetic assays**

Pharmacokinetic sampling will be done after at least 5 days of rifabutin-containing anti-mycobacterial therapy without (Group I) or with (Group II) HAART. Blood samples will be collected just before rifabutin administration and then 0.5, 1, 2, 4, 6, 8 and 24 hours afterward. Patients of Group II treated with 150 mg of rifabutin every other day will undergo additional sampling at 48 hours.

The plasma concentrations of rifabutin and its major metabolite, 25-*O*-desacetyl rifabutin will be determined simultaneously by validated high-pressure liquid chromatography (HPLC). Blood samples will be taken in heparin-containing tubes, placed on ice and centrifuged at 3000 x *g* for 10 min. Then, the obtained plasma will be deproteinized by using three times volume of methanol and centrifuged 15,000 x *g* for 5 min, and the supernatant will be used for assay.

The area under the curve (AUC) will be calculated using non-compartmental techniques (WinNonlin, ver. 5, Pharsight Corp., Mountain View, CA) based on the obtained values (AUC 0-24 h for all, AUC 0-48 h for Group II). The maximum plasma concentration (C_max_) and time of C_max_ (T_max_) will be determined directly from the data.

**4.7 Statistical analysis**

Differences between groups were determined by using the Fisher’s exact test for categorical data and the Mann Whitney’s test for continuous variables. For all statistical analyses, differences were considered significant if the *p* value was less than 0.05.

**5. ETHICAL CONSIDERATIONS**

**5.1 Ethics Committee**

This study will be conducted in accordance with the ethical principles that have their origin in the Declaration of Helsinki and applicable regulatory requirements that prescribed in "Ethics Guidelines for Clinical Research" (July 30, 2003, Ministry of Health, Labor and Welfare) in Japan. Prior to the study, the investigator must have written and dated approval opinion from the IRB/EC of all of the NCGM for the protocol, consent form and any other written information to be provided to subjects.

**5.2 Informed Consent**

Investigators must ensure that participants are clearly and fully informed about the study. Participants will be told that they are free NOT to participate in the study and reassured that this will not affect their care in any way.

**5.3 Confidentiality**

Data obtained from participants during study shall be treated with absolute confidentiality. Individual names, chart numbers and addresses are omitted from Case report form (CRF) and questionnaires and the unique codes will be used in the study. CRFs will be stored in a locked cabinet at ACC office and will be destroyed when the study is completed. When the results of the study will be published at the academic conferences or in the scientific journals, privacy of the participants is completely protected.

**6. ADMINISTRATION SECTION**

**6.1 Compliance with the Protocol**

This study shall be conducted in this approved protocol. The investigator should not implement any deviation or change to the protocol without prior review and documented approval/favorable opinion from the IRB/EC of an amendment.

**6.2 Records and Reports**

Investigators are required to prepare and maintain adequate and accurate case histories to record all observations. Documents must be consistent with the source documents or discrepancies must be explained.

**6.3 Budget**

This study will be financially supported by the Health Labour Sciences Research Grant (#H18-AIDS-008) from the Ministry of Health, Labour and Welfare, Government of Japan.

The cost for the examination of blood concentration level of rifabutin stated in the protocol will be covered within the research fund but not the cost of rifabutin and routine laboratory examination will not.

**7.1 List of researchers**

Primary investigator:

Junko Tanuma, M.D., AIDS Clinical Center (ACC), National Center for Global Health and Medicine (NCGM)

Other investigator:

Katsuji Teruya, M.D., ACC, NCGM

Koji Watanabe, M.D., ACC, NCGM

Takahiro Aoki, M.D., ACC, NCGM

Haruhito Honda, M.D., ACC, NCGM

Hirohisa Yazaki, M.D., ACC, NCGM

Kunihisa Tsukada, M.D., ACC, NCGM

Hiroyuki Gatanaga, M.D., ACC, NCGM

Yoshimi Kikuchi, M.D., ACC, NCGM

Shinichi Oka, M.D., ACC, NCGM

Kazumi Sano, Ph. Department of Drug Metabolism and Disposition, Meiji Pharmaceutical University, Tokyo, Japan
