# Supplementary material for: Pharmacokinetics of Rifabutin in Japanese HIV-Infected Patients with or without Antiretroviral Therapy
Source: PLoS One. 2013 Aug 5;8(8):e70611. doi: 10.1371/journal.pone.0070611 (PMC3734254; doi:10.1371/journal.pone.0070611)
Supplement: Protocol S1 — Protocol and IC form in Japanese. The full version of the study protocol and the informed consent form in Japanese. (PDF) [file pone.0070611.s002.pdf]

よくお読みください

臨床試験への参加を考えている皆さんへ

説明文書および参加同意書

試験課題名：
日本人HIV感染者を対象としたリファブチンの薬物動態の検討を目的とした臨床薬理試験

内容についてわからないことや聞きたいことがありましたら，
いつでも遠慮なくお申し出ください。

国立国際医療センター　エイズ治療研究開発センター

第１版　2007年12月19日

リファブチン血中濃度に関する臨床試験への参加を考えている方へ

これから，あなたにご協力いただく試験の内容について説明いたします。

この臨床試験に参加するかどうかはあなたの自由意思によります。以下の内容について説明を受け，十分理解した上で，参加してもよいと思われた場合は、同意書に署名（しょめい）してください。

1．はじめに

今回血中濃度を測定するリファブチンという薬は，結核または非結核性抗酸菌症（結核菌以外の抗酸菌による感染症）を治療する薬で，すでに日本で用いられているリファンピシンと同じ系統の薬です。リファブチンはリファンピシンに比べると，薬物相互作用（他の薬と併せて飲んだときに，リファブチンまたは併せて飲んだ薬の血中濃度が上下すること）が弱いことが知られています。一方，エイズの治療に使われる薬の多くは，リファンピシンと併せて服用したときに薬物相互作用を示すことから，リファンピシンと併せて服用することは禁止されています。これに対し，リファブチンは服用する薬のカプセルの数を調節すれば，エイズの治療薬と併せて服用することができます。

しかし，患者様の状態や服用しているエイズ治療薬の種類によって，リファブチンの血中濃度は大きく変化することが、徐々に分かってきました。実際、2007 年の国際エイズ会議では、HIV 患者様では有効なリファブチンの血中濃度が得られていないことがある、という報告がありました。

こういった背景をもとに、今後の結核または非結核性抗酸菌症治療に役立てるため、今回、HIV 患者様にリファブチンを服用して頂いた際の，リファブチン服用時の血中濃度を詳しく調査することにしました。

なお、この臨床試験は，治験審査委員会（ちけんしんさいいんかい）（臨床試験が安全かつ適切に行われるかを審査する当クリニックの委員会）において，臨床試験に参加される方の権利が守られ，倫理的かつ科学的に問題ないことが調査，審議され，承認を受けた上で，行われます。

2．リファブチンの概要

一般名：リファブチン

商品名：Mycobutin 150 mg カプセル

リファブチンは，現在，結核の治療に用いられているリファンピシンと同じ系統の薬で，外国では結核だけでなく，非結核性抗酸菌症（結核菌以外の抗酸菌による感染症）の治療にも用いられています。

2001 年１０月現在，世界５４か国で承認されています。日本では承認されていませんが，HIV に感染した結核または非結核性抗酸菌症の患者様には、抗 HIV 薬との併用療法において必要な薬であることから、国立国際医療センター・エイズ治療研究開発センターまたは厚生労働省エイズ治療薬研究班[注1)]（班長　東京医科大学　臨床検査医学科　主任教授　福武　勝幸）において個人輸入され、診療に用いられています。

３．臨床試験の流れ

1.　対象となる方

この臨床試験では，結核または非定形抗酸菌症の治療として、リファブチンを服用する HIV 患者様を対象としています。

ただし、以下の基準に該当される場合，この臨床試験へ参加できません。

１）リファブチンに対する薬物アレルギーの既往歴を有する方
２）投与開始日から投与前 28 日までの間でリファブチンを服用された方（第１群のみ）
３）血液検査の結果、AST（GOT），ALT（GPT）が 200IU/L 以上、総ビリルビンが 5.0mg/ml 以上、推定クレアチニンクリアランスが 30ml/分未満、の方
４）その他主治医が適切でないと判断した方

2.　服用のスケジュール

この臨床試験では、２種類の服用方法におけるリファブチンの血中濃度を調べます。

第１群:リファブチン 150 mg カプセル１日１回２カプセルずつ服用
＊抗 HIV 療法を受けていない方が対象です。

第２群:リファブチン 150 mg カプセル１日１回１カプセル毎日または１日おきに服用
＊プロテアーゼ阻害剤を含む抗 HIV 療法の併用が必要な方が対象です。

検査のスケジュール

リファブチン血中濃度の測定は，設定された服用方法が開始された日，1 週目（7 日目～14 日）および１ヶ月目（20 日目～40 日目）に，行います。

それぞれ，服用前， 30 分後，1，2，4，6，8，12，24，48 時間後に１回３ mL の採血をします。

検査が始まる前に、日時など、検査スケジュールを書いた表をお渡し致します。ご入院中の方は、担当医が採血に参りますので、病室でお待ちください。外来にお越しの場合は、ACC 外来で看護師または医師が採血致しますので、各採血時刻になったら、ACC 外来までお越しください。

注[1)] 厚生労働省エイズ治療薬研究班（http://www.iijnet.or.jp/aidsdrugmhw/org.html/）：日本で未承認もしくは該当する適応症が未承認であるが，海外では目的とする疾患の治療ために既に承認されている薬剤を，主任研究者（班長）が医師個人輸入として輸入し，当該薬剤を必要とする患者の担当医師の要請に応じて，治療研究のために無償で交付し，治療効果，安全性，副作用などを明確にするとともに，将来，国内での薬剤の入手難を緩和することを目的としている。

血中濃度以外の検査項目

健康状態や副作用の有無を確認するため，血中濃度以外にも、服用前と，治験の最後に下記に示す血液検査（約 10 mL 採血）を実施します。この他にも担当医師が必要と判断した場合には，追加検査のための採血を行うことがありますのでご了承下さい。

① 血液学的検査（貧血，出血傾向，血液疾患，炎症の有無等を調べます）：
白血球数，白血球分画（好中球，好酸球，好塩基球，リンパ球，単球），ヘモグロビン，ヘマトクリット，赤血球数，血小板数

② 血液生化学的検査（肝臓・胆道系，腎臓，代謝機能等について調べます）：
AST（GOT），ALT（GPT），アルカリフォスファターゼ，LDH，総ビリルビン，アルブミン，クレアチニン，尿酸，カルシウム（Ca^{++}），ナトリウム（Na^{+}），カリウム（K^{+}），

③ 尿検査（主に腎臓，泌尿器系について調べます）：
pH，蛋白（定性），糖（定性），潜血（定性）

４．臨床試験への参加予定期間と参加予定人数

この臨床試験は，2008年２月から 2009年３月までの間に行われ、約 20 名の患者様が参加する予定です。

5. 予期される利益または不利益

リファブチンは国内未承認薬ですが，HIV に感染した結核・非結核性抗酸菌症の治療には抗 HIV 薬との併用療法において欠かせない薬であることから、エイズ治療研究開発センターでは、個人輸入して診療に用いています。本治験では、治療上の必要でリファブチンを服用している方を対象にしているため、本治験に参加することで得られる治療上の利益や不利益はありません。また、万が一，この臨床試験中あるいは臨床試験終了後に，リファブチンによる健康被害が生じた場合には，通常の診療と同様、適切な診察と治療を受けることができます。

一方、リファブチンは，外国では，エイズ患者におけるMAC（マイコバクテリウム・アビウムコンプレックス）症の発症予防，非結核性抗酸菌症（エイズ患者または非エイズ患者）または結核の治療に承認されて，有効性および安全性が検証されてきました。リファブチンの米国添付文書に基づく副作用情報を、以下に示します。

リファブチン投与患者に1%以上の頻度で見られた有害事象		
有害事象	リファブチン（566例）%	プラセボ（580例）%
腹痛	4	3
無力症	1	1
胸痛	1	1
発熱	2	1
頭痛	3	5
疼痛	1	2
食欲不振	2	2
下痢	3	3
消化不良	3	1
おくび	3	1
鼓腸放屁	2	1
嘔気	6	5
嘔気・嘔吐	3	2
嘔吐	1	1
筋肉痛	2	1
不眠	1	1
皮疹	11	8
味覚倒錯	3	1
尿の変色	30	6

1%未満の頻度で見られた有害事象としては、以下があります。

- インフルエンザ様症候群，肝炎，溶血，関節痛，筋炎，呼吸困難をともなう胸部圧迫感または胸痛，皮膚の変色

リファブチンが原因であるか不明ですが、1例以上に以下の有害事象が見られました。

- 発作，知覚異常，失語症，錯乱，心電図上の非特異的なT波変化

臨床検査値の異常変動と考えられる臨床検査値の変化

臨床検査値の異常変動が見られた患者の比率		
臨床検査値の異常変動	リファブチン(566 例)%	プラセボ（580 例）%
化学検査：		
AlP の上昇[1]	＜1	3
SGOT の上昇[2]	7	12
SGPT の上昇[2]	9	11
血液学的検査：		
貧血[3]	6	7
好酸球増加	1	1
白血球減少[4]	17	16
好中球減少[5]	25	20
血小板減少[6]	5	4

グレード 3 または 4 の変化（下記の基準による）
1：すべての測定値が＞450U/L
2：すべての測定値が＞150U/L
3：すべてのヘモグロビン測定値が＜8.0g/dL
4：すべての WBC 測定値が＜1,500/mm^3
5：すべての ANC 測定値が＜750/mm^3
6：すべての血小板数測定値が＜50,000/mm^3

また、フルコナゾールおよび（または）マクロライド系抗生物質と併用した場合，ぶどう膜炎（眼球を構成する膜の一部の炎症）の頻度が高くなったという報告があります。

このような外国の臨床試験で、服用中止に至った有害事象の頻度は，リファブチン群で 16%，プラセボ投与群で 8%でした。その主な理由は，皮疹（投与患者の 4%），消化管障害（3%），好中球減少症（2%）でした。

6．この臨床試験への参加はあなたの自由意思によるものであり，いつでも取りやめることができます

この臨床試験に参加するかどうかはあなたの自由です。参加することに同意した後でも，または，臨床試験薬の服用を開始した後でも，あなたが止めたいと思ったときはいつでも止めることができます。参加を断ってもあなたが不利益を受けることは一切ありません。リファブチンの服用は、あくまでも治療上の必要性に応じて決定されます。

7．あなたの臨床試験への参加の継続に影響を与える可能性のある情報は，速やかにあなたに伝えられます

この臨床試験に関連して、新たに重要な情報が見つかった場合には，速やかに担当医師がご連絡致します。それにより臨床試験への参加を止めたい場合は，申し出てください。

8.臨床試験への参加を中止する場合の条件または理由

臨床試験への参加に同意した後に、病状が変化した場合や、リファブチンが中止された場合など，担当医師が臨床試験への参加・継続が好ましくないと判断したときは，臨床試験への参加を中止していただくことがあります。

9．プライバシーの保護について

この臨床試験の結果（同意を撤回（てっかい）した場合など途中で臨床試験を中止した場合でも，その時までの結果）は、きちんと記録されます。また，当院の臨床試験審査委員会および厚生労働省などの規制当局が，この記録が正確であるかどうかを確認するため，あなたの記録（あなたの氏名，住所を含む）を閲覧（えつらん）することがあります。得られたデータは医学雑誌や学会等に発表されたり、医薬品の安全性や有効性に関する情報として使用されたりしますが、その場合でも個人が特定できるような情報は一切公開されず，プライバシーは守られます。この同意書に署名していただくことにより、あなたから結果の公表について許可を得たこととさせていただきます。

すべての関係者には、秘密を守る義務が課せられており，あなたの個人情報が漏れることがないように個人データを保護するために厳重な対策をとっています。

なお，あなたが臨床試験に参加された後に，同意を撤回された場合や中止された場合でも，その時までのデータは，同様に使用されます。

10．担当医師の氏名，および連絡先

施　設　名　：国立国際医療センター　エイズ治療研究開発センター
試験責任医師　：田沼　順子
臨床試験相談窓口　：

〒162-8655　東京都新宿区戸山 1-21-1
国立国際医療センター　エイズ治療・研究開発センター　田沼順子
TEL: 03-3202-7181　FAX: 03-3207-7198
受付時間　9：00～17：30（平日・土・日・祝日）
夜間，土・日・祝日，緊急時は、当直医が対応します。

ご不明な点がありましたら，主治医または上記窓口まで、遠慮なく申し出てください。

同　意　書

施設保管用

臨床試験課題名：
日本人 HIV 感染者におけるリファブチンの薬物動態の検討を目的とした臨床薬理試験

私は臨床試験参加に先立ち，試験薬の概要，今回の臨床試験の目的・内容および臨床試験関連の諸条件について，国立国際医療センター内において説明文書を用いて説明を受け，質問する機会と十分な時間が与えられました。その内容を承知した上でこの臨床試験に参加することに同意します。

なお，本同意書は２部作成され，１部を私の控えとして受け取りました。

- 同意は私の自由意志に基づくものであり，いつでも取り消すことができ，取り消した場合でも私への対応や治療において不利な扱いを受けないこと。
- この臨床試験を調査審議した臨床試験審査委員会および規制当局の関係者により私の医療記録が見られることがあること。
- この臨床試験から得られた情報は，医薬品の安全性や有効性に関する情報として使用されること。また，同意を撤回した場合や中止した場合でもその時までの情報は，同様に使用されること。
- 個人を特定できるような情報は公表されないこと。

説明日　　　　　　　　　説明者：所属　　　　　医師名

　　年　　月　　日　＿＿＿＿＿＿＿＿＿＿　＿＿＿＿＿＿＿＿＿＿

同 意 年 月 日　　　　　年　　月　　日

被験者氏名＿＿＿＿＿＿＿＿＿＿＿＿＿＿＿＿

同　意　書

被験者控え用

臨床試験課題名：
日本人 HIV 感染者におけるリファブチンの薬物動態の検討を目的とした臨床薬理試験

私は臨床試験参加に先立ち，試験薬の概要，今回の臨床試験の目的・内容および臨床試験関連の諸条件について，国立国際医療センター内において説明文書を用いて説明を受け，質問する機会と十分な時間が与えられました。その内容を承知した上でこの臨床試験に参加することに同意します。

なお，本同意書は２部作成され，１部を私の控えとして受け取りました。

- 同意は私の自由意志に基づくものであり，いつでも取り消すことができ，取り消した場合でも私への対応や治療において不利な扱いを受けないこと。
- この臨床試験を調査審議した臨床試験審査委員会および規制当局の関係者により私の医療記録が見られることがあること。
- この臨床試験から得られた情報は，医薬品の安全性や有効性に関する情報として使用されること。また，同意を撤回した場合や中止した場合でもその時までの情報は，同様に使用されること。
- 個人を特定できるような情報は公表されないこと。

説明日　　　　　　　　　　説明者：所属　　　　　医師名

　　年　　月　　日　　　______________　　______________

同 意 年 月 日　　　　　　年　　月　　日

被験者氏名______________________________

日本人HIV感染者における
リファブチンの薬物動態の検討を目的とした
臨床薬理試験

試験実施計画書

研究代表者
国立国際医療センターエイズ治療研究開発センター
田沼　順子

研究協力者
国立国際医療センター　エイズ治療・研究開発センター
岡　慎一　センター長
菊池　嘉　部長
立川　夏夫　情報室長
照屋　勝治　5階南病棟医長
潟永　博之　専門外来医長
本田　美和子　技官
塚田　訓久　技官
矢崎　博久　技官
本田　元人　技官
渡邉　珠代　専門修練医
神村　麻穂子　レジデント
渡辺　恒二　レジデント
柳沢　邦雄　レジデント
仲村　秀太　レジデント
後藤　耕司　レジデント
国立国際医療センター　薬剤部
佐野　和美　研修生

試験実施計画書の概要

1. 表　題

日本人HIV感染者におけるリファブチンの薬物動態の検討を目的とした臨床薬理試験

2. 目　的

1）主要目的

日本人HIV感染者を対象に, リファブチンを投与した時のリファブチンの薬物動態を検討する。

2）副次目的

日本人HIV感染者のリファブチンと抗レトロウイルス薬との薬物動態的な相互作用について検討する。

3. 対　象

結核・非定型抗酸菌症に対しリファブチンを服用する日本人 HIV 感染者で，本試験への参加を本人の自由意思により同意した者。

4. 試験薬

ミコブティン^{TM} 150 mg カプセル： 1カプセル中にリファブチン 150 mg を含有する。

5. 試験方法

1）試験デザイン

前向き非盲検化試験。

2）対象

第1群：抗レトロウイルス薬非併用群
第2群：抗レトロウイルス薬併用群

投与群	被験者		試験日		
	例数	プロテアーゼ阻害剤を含むHAART	1日目	1週目（第7-14日）	1ヶ月目（第20日～第40日）
第1群	10 例	無し	300mg 1日1回		
第2群	10例	有り	150 mg 1日1回連日または隔日		

3）投与量・投与方法

第1群：リファブチン 300mg を1日1回空腹時に投与する。
第2群：リファブチン 150 mg を1日1回連日または隔日空腹時に投与する。

*第2群のリファブチン投与量は、「Guidelines for the Use of Antiretroviral Agents in HIV-1-Infected Adults and Adolescents - December 1, 2007」米国DHHS: A Working Group of the Office of AIDS Research Advisory Council (OARAC) に基づいて調節する。

6. 観察・検査項目

血漿中薬物濃度

被験者背景，自覚症状，身体所見（医師の診察，身長，体重含む）

7. 評価項目

1）薬物動態

血漿中薬物動態パラメータ（リファブチン）：

１日目，１週目および１ヶ月目の C_{max}，T_{max}， AUC_{τ}，および R_{ac}，また可能なら $t_{1/2}$

2）安全性

自覚症状・他覚所見，臨床検査値異常の有無，その他有害事象

8. 目標被験者数

20 例（各群 10 例）

9. 試験実施期間

2008 年 2 月 12 日 ～ 2009 年 3 月 31 日

目次

1 序文 19

2 目的 20

3 試験デザイン 21

4 被験者選択・除外基準 21

4.1 選択基準 21

4.2 除外基準 21

5 試験薬 21

5.1 試験薬の説明および包装形態 21

5.2 試験薬の投与 24

5.3 併用薬および併用療法 24

6 試験の実施の手順 24

6.1 スクリーニング 24

6.2 被験者の登録 20

6.3 試験期間 20

6.3.1 投与前 20

6.3.2 第 1 日目 21

6.3.3 第 7～14 日目 21

6.3.4 第 20～40 日目 21

6.4 被験者の中止基準と手順 21

7 評価 21

7.1 血漿中 リファブチン濃度の測定 21

7.2 その他の臨床検査 22

8 有害事象の報告 22

8.1 有害事象 22

8.2 報告期間 22

8.3 検査値異常 22

8.4 重篤な有害事象 23

8.5 重症度評価 23

9 統計解析 23

9.1 症例数の設定 23
9.2 薬物動態解析 24
9.2.1 血漿中濃度 24
9.2.2 薬物動態パラメータ 15
10 品質管理および品質保証 24
11 データの取扱いおよび記録の保存 25
11.1 症例報告書・電子的データ 25
12 倫理 25
12.1 試験倫理委員会 25
12.2 被験者への情報および同意 25
13 試験依頼者が試験を中止する際の基準 25
14 結果の公表 25
15 研究事務局 30
16 参考資料 30

1. 序文

リファブチン（Rifabutin：RBT）は，リファンピシン（Rifampicin：RFP）を改良したリファンピン系抗菌薬であり，結核菌を含む各種抗酸菌に対する優れた抗菌活性のみならず，理想的な薬物動態および組織分布を有する抗菌薬である。また，CYP3A4 に対する影響は RFP に比べると弱く，HIV 感染症治療薬として用いられる各種プロテアーゼ阻害薬などとの併用が可能である[注2)]。

外国で公表されている資料[i, ii] に基づくと，リファブチンの臨床的特徴は以下のように要約される。

薬物動態

リファブチン経口投与後の薬物動態的特徴は以下のとおりであった。

- 健康成人にリファブチン 300～600 mg を経口投与した場合の C_{max} は 0.4～0.7 μg/mL, $t_{1/2}$ は 35～40 時間であった。
- リファブチンは，脳を除く種々の組織に広範に移行し，肺，胆汁，胆嚢，小腸壁では，血漿中濃度を上回る組織内濃度を示した。
- 5 種類の代謝体が認められるが，主なものは，25-O-deacetyl 誘導体と 31-hydroxyl 誘導体である。特に 25-O-deacethyl 誘導体はリファブチンと同程度の抗菌活性を示した。
- リファブチンおよびその代謝体は，尿から排泄された。

有効性

- ヒト免疫不全ウィルス（Human Immunodeficiency Virus：HIV）陽性患者における播種性 *Mycobacterium avium* Complex（MAC）症発症抑制：リファブチン 300 mg は，CD4 陽性リンパ球数が 200 /μL 以下の HIV 陽性患者における播種性 MAC 症の発症抑制作用を示した。
- 非結核性抗酸菌症（NonTuberculous Mycobacteriosis：NTM）症（HIV 陽性患者，HIV 非感染者）：リファブチン（450～600 mg）を含む 2～3 剤併用療法（イソニアジド：INH，クロファジミン[注3)]，エタンブトール：EB）は，HIV 陽性患者および HIV 非感染者における NTM 症に対し，治療効果を示した。
- 結核
  - 初回治療例：リファブチン（150～300 mg）を含む 2～3 剤併用療法（INH，EB，ピラジナミド：PZA）は，結核初回治療例に対し，治療効果を示した。
  - 多剤耐性例：抗結核第一選択薬に対して耐性を示す多剤耐性結核に対し，リファブチン（300, 450 mg）を含む 2～3 剤併用療法は有効であった。

安全性

- リファブチン 150～600 mg 投与時の副作用は消化器系，血液およびリンパ系障害であり，RFP 投与時と同様であった。いずれも忍容できるものであった。

注2）リファブチン USPI（http://www.iijnet.or.jp/aidsdrugmhw/text/2kousei/d/Myco/2_d_Myco.htm）
注3）本邦ではハンセン病治療薬として承認されているが，抗結核薬としては承認されていない。

外国では，米国を始めとして世界 54 カ国・地域において承認されている（2001 年 10 月現在[注4]）。本邦では，リファブチンは未承認薬であるが，現在，厚生労働省エイズ治療薬研究班[注5]（班長　東京医科大学　臨床検査医学科　主任教授　福武　勝幸）または国立国際医療センターエイズ治療・研究開発センターにおいて個人輸入され，国内エイズ患者の治療に用いられている。

本邦における結核を含む抗酸菌症治療の現状には，数多くの解決すべき問題点がある。そのうちの一つとして，非定形抗酸菌症の治療に用いることの出来る薬剤は，わずかに HIV 感染者における MAC 症の発症予防に 1 剤（アジスロマイシン：AZM），MAC 症の治療に 1 剤（クラリスロマイシン：CAM）のみしかないことがあげられる。また抗結核薬については，いずれも 30 年以上前に開発された薬剤のみである。諸外国の結核および NTM 症の治療にはリファブチンが標準的に用いられている [1, iii, iv, v, vi]。

一方、リファブチンは RFP に比べると，CYP3A4 をはじめとする酵素に対する誘導作用は弱く，種々の抗 HIV 薬との併用が可能であるが，抗 HIV 薬とリファブチンを併用した場合のリファブチンの血中濃度については，十分検討されておらず，十分な抗抗酸菌活性が得られているかどうか，不明である。実際，2007 年国際エイズ会議において，HIV 結核患者ではリファブチンの単独投与およびプロテアーゼ阻害剤と併用投与した際の AUC が，薬剤耐性獲得基準値や治癒基準値を下回っていたという報告がなされている [7), 8)]。

また，人種間の違いによる薬物動態の変化は，これまで全く検討されておらず、アジア人における薬物動態のデータは皆無である。過去に，エイズ治療・研究開発センターにおいて、本人の同意のもとリファブチン血中濃度を測定した HIV 患者は 3 名いるが，それぞれ症例①リファブチン 300mg 投与 24 時間後血中濃度 0.136μg/mL，症例②ロピナビル・リトナビル併用下リファブチン 150mg 投与 25 時間後 0.398μg/mL，症例③リファブチン 300mg 投与（測定時刻不明）0.301μg/mL と，測定値にはおおきなばらつきがみられ，また，単回測定では薬剤耐性獲得基準値や治癒基準値に達しているか判断ができないことから，計画的な薬物動態試験の必要性が再確認された。

そこで，現在，リファブチンを治療に用いている日本人 HIV 感染者において、リファブチンの薬物動態を検討することとした。

2. 目的

1）主要目的

日本人 HIV 感染者を対象に，リファブチンを投与した時のリファブチンの薬物動態を検討する。

注[4]）ファイザー株式会社情報
注[5]）厚生労働省エイズ治療薬研究班（http://www.iijnet.or.jp/aidsdrugmhw/org.html/）：日本で未承認もしくは該当する適応症が未承認であるが，海外では目的とする疾患の治療ために既に承認されている薬剤を，主任研究者（班長）が医師個人輸入として輸入し，当該薬剤を必要とする患者の担当医師の要請に応じて，治療研究のために無償で交付し，治療効果，安全性，副作用などを明確にするとともに，将来，国内での薬剤の入手難を緩和することを目的としている。

2）副次目的

日本人HIV感染者のリファブチンと抗レトロウイルス薬との薬物動態的な相互作用について検討する。

3. 試験デザイン

結核・非定型抗酸菌治療に対しリファブチンを服用中の日本人HIV感染者を対象にした、前向き非盲検試験である。抗レトロウイルス薬非併用群（第1群）と，抗レトロウイルス薬併用群（第2群），それぞれにおいて，薬物動態を検討する。

【試験デザインの設定根拠】

本試験は治療中の患者における薬物動態の検討を主要目的とするため，被験者全員に実薬を投与する非盲検試験とした。また，抗レトロウイルス薬の有無によりリファブチン投与量が異なることや，抗レトロウイルス薬との相互作用が想定されるため，抗レトロウイルス療法の有無により患者群を分けた。

4. 被験者選択・除外基準

4.1 選択基準

結核・非定型抗酸菌に対する治療として，リファブチンを服用している日本人HIV感染者で，本試験への参加を本人の自由意思により同意した者のうち，以下の基準を満たす者。

1. 年齢が20歳以上の日本人であること。
2. 本試験に関する必要な情報について，本人が説明を受け，本試験への参加の意思を同意文書（署名および日付が記入されたもの）により確認できる者
3. 予定された来所，投薬スケジュール，臨床検査およびその他の試験の手順を遵守する意思があり，かつ遵守することができる者

4.2 除外基準

以下のいずれかに該当する被験者は試験に組み入れない。

1. リファブチンに対するアレルギーの既往歴を有する者
2. 第1群については、試験薬の投与前28日以内に，リファブチンを服用している者
3. ALT/ASTが共に250 IU/L 以上の者
4. T.bil が5.0 mg/dl以上の者
5. クレアチニンクリアランスが30ml/分未満の者
6. その他主治医がこの臨床試験への参加が適切でないと判断する者

5. 試験薬

5.1 試験薬の説明および包装形態

本臨床試験で使用するMycobutin 150 mg カプセルは，紫色の粉末の入った濃赤色のカプセル（0号）である。Mycobutin カプセルは，1カプセルあたりリファブチン150 mgを含有する（高密度ポリエチレ

ン瓶，100 カプセル/瓶）。

試験薬の副作用

外国で実施された臨床試験でリファブチンを服用した時の副作用を以下に示す。この試験で有害事象のため，投与中止となった患者数は，リファブチン群で 16%，プラセボ投与群で 8%であった。リファブチンの投与中止に至った主な理由は，皮疹（投与患者の 4%），消化管障害（3%），好中球減少症（2%）であった。

リファブチン投与患者に 1%以上の頻度で見られた有害事象		
有害事象	リファブチン（566 例）%	プラセボ（580 例）%
腹痛	4	3
無力症	1	1
胸痛	1	1
発熱	2	1
頭痛	3	5
疼痛	1	2
食欲不振	2	2
下痢	3	3
消化不良	3	1
おくび	3	1
鼓腸放屁	2	1
嘔気	6	5
嘔気・嘔吐	3	2
嘔吐	1	1
筋肉痛	2	1
不眠	1	1
皮疹	11	8
味覚倒錯	3	1
尿の変色	30	6

リファブチン投与患者の 1%未満の発現頻度で，以下の有害事象が起こる可能性がある。

- インフルエンザ様症候群，肝炎，溶血，関節痛，筋炎，呼吸困難をともなう胸部圧迫感または胸痛，皮膚の変色。

リファブチン投与患者の 1 例以上の発現頻度で，以下の有害事象が見られたが，その原因は不明である。

- 発作，知覚異常，失語症，錯乱，心電図上の非特異的な T 波変化。

リファブチンを 1050～2400 mg/day で投与したときに全身の関節痛およびぶどう膜炎が報告された。これらの有害事象はリファブチンの投与中止により軽快した。

臨床検査値の異常変動と考えられる臨床検査値の変化

臨床検査値の異常変動が見られた患者の比率		
臨床検査値の異常変動	リファブチン（566 例）%	プラセボ（580 例）%
生化学検査		
AlP の上昇 [1]	＜1	3
SGOT の上昇 [2]	7	12
SGPT の上昇 [2]	9	11
血液学的検査		
貧血 [3]	6	7
好酸球増加	1	1
白血球減少 [4]	17	16
好中球減少 [5]	25	20
血小板減少 [6]	5	4

グレード 3 または 4 の変化（下記の基準による）
1：すべての測定値が＞450U/L
2：すべての測定値が＞150U/L
3：すべてのヘモグロビン測定値が＜8.0g/dL
4：すべての WBC 測定値が＜1,500/mm^3
5：すべての ANC 測定値が＜750/mm^3
6：すべての血小板数測定値が＜50,000/mm^3

好中球減少発現率は，プラセボ投与群に比べ，リファブチン投与群のほうが有意に高い結果であった（p＝0.03）。一方，血小板減少症発現頻度は，有意な結果ではなかった。しかし，まれではあるが，リファブチンが血小板減少を引き起こす可能性がある。外国で実施された臨床試験では，HIV 感染者の 1 例で，リファブチンに起因する血栓性血小板減少性紫斑病が発現した。

HIV 感染者における MAC 症発症予防を目的に，リファブチンを 300 mg/日で単独投与した場合，ぶどう膜炎はほとんど起こらなかった。また，フルコナゾールおよび（または）マクロライド系抗生物質と併用した場合も同様であった。しかし，300 mg/日以上のリファブチンをフルコナゾールおよび（または）マクロライド系抗生物質と併用した場合，ぶどう膜炎の発現率は高くなった。なお，リファブチン投与時にみられたぶどう膜炎の重症度は軽度～重度であったが，コルチコステロイドおよび散瞳点眼薬あるいは一方の投与で軽快した。ただし，重症例の中には，症状の軽快までに数週間を要した症例もある。ぶどう膜炎が起こった場合，リファブチンを一時中止し，眼科学的検査を実施することが推奨されてい

る。軽症例のほとんどではリファブチンの投与再開が可能であるが，徴候または症状が再発した場合には，リファブチンの投与を中止する必要がある。
（リファブチン米国添付文書 http://www.iijnet.or.jp/aidsdrugmhw/text/2kousei/d/Myco/2_d_Myco.htm より引用）

5.2　試験薬の投与

試験協力者は被験者に約 150 mL の常温の水と共に試験薬を投与する。薬物濃度測定を対象とした検体の採取条件を一定にするため，投与後 4 時間は，なるべく臥床しないよう促す。

投与量は以下のとおりである。

第 1 群：リファブチン 300mg を 1 日 1 回空腹時に投与する。

第 2 群：リファブチン 150 mg を１日 1 回連日または隔日空腹時に隔日投与する。

*第2群のリファブチン投与量は、「Guidelines for the Use of Antiretroviral Agents in HIV-1-Infected Adults and Adolescents - December 1, 2007」米国DHHS: A Working Group of the Office of AIDS Research Advisory Council (OARAC) に基づいて調節する。

5.3　併用薬および併用療法

試験期間中に投与された試験薬以外のすべての薬剤について，適応症，1 日量，投薬開始日および終了日を記録する。すべての被験者に対して来院時に併用薬の有無を確認する。

試験薬の投与開始前 28 日以内に何らかの薬剤が使用された場合，「前治療薬」として記録する。

6. 試験の実施の手順

6.1 スクリーニング

試験薬の投与前 28 日以内に被験者のスクリーニングを行い，被験者が本試験で規定された基準を満たしていることを確認する。試験責任（分担）医師は 12.2.項「被験者への情報および同意」に従って各被験者から同意を得る。

6.2 被験者の登録

試験責任医師は，本試験のスクリーニングを実施した被験者に対し，順にスクリーニング番号を割り付ける

6.3 試験期間

6.3.1 投与前

試験薬投与前までに実施する手順を以下に示す。

被験者の選択基準・除外基準にに合致しないか確認

試験責任（分担）医師による身体的検査・体重測定

試験薬投与前に服用した薬剤または併用薬の確認

前治療法および併用療法の確認
スクリーニング以降，被験者の病歴に変更がないか確認
臨床検査用の検体採取（7.2 項「その他の臨床検査」参照）

6.3.2 第 1 日目

試験責任（分担）医師による身体的検査
併用薬および併用療法の確認，および有害事象の確認
薬物濃度測定のための採血　（7.1 項「薬物動態」参照）

6.3.3 第 7～14 日目

試験責任（分担）医師による身体的検査
併用薬および併用療法の確認，および有害事象の確認
薬物濃度測定のための採血　（7.1 項「薬物動態」参照）

6.3.4 第 20～40 日目

試験責任（分担）医師による身体的検査
併用薬および併用療法の確認，および有害事象の確認
薬物濃度測定のための採血　（7.1 項「薬物動態」参照）

6.4 被験者の中止基準と手順

試験は被験者の希望で，あるいは試験責任医師の判断で安全性，被験者の行動，事務的な理由で随時中止することができる。被験者が規定の来院日に来院しなかった場合，あらゆる努力を払って被験者と連絡を取る。いずれの場合も被験者の転帰を可能な限り記録する。また，該当する場合，未回復の有害事象に関して被験者の経過観察を依頼しなくてはならない。

被験者が試験を中止し，将来の情報開示に関する同意も撤回する場合，さらなる評価の実施および追加データの収集は行わない。試験責任（分担）医師は同意撤回前に収集したデータを保持し，引き続き使用することができる。

脱落症例の補充は，試験責任（分担）医師が必要と判断した場合に行う。

7. 評価

7.1　血漿中 リファブチン濃度の測定

以下のスケジュールで薬物濃度測定のための採血をおこなう。

血液検体はヘパリン入り採血管を用いて採血する。各時点での採血量は，3mL である。

＜採血時期＞

第 1 群：第 1 日，1 週間目，1 ヶ月目
投与前，投与後 0.5，1，2，4，6，8，12，24 時間後

第 2 群：第 1 日，1 週間目，1 ヶ月目
投与前，投与後 0.5，1，2，4，6，8，24，(隔日投与の場合に限り)48 時間後

各投与期の試験実施計画書で規定された各時間に，3mL 採血し，血漿を全て採取する。なお，検体の採取には適切にラベルを貼付したヘパリンナトリウム入り採血管を用いる。実際の採血時間を変更することは可能であるが，採取する検体数は同じでなければならない。ただし，正確な採血時間が原資料および症例報告書上に記載され，予定採血時間の 10%以内（例えば投与後 60 分の採血であれば 6 分以内）に採血が行われていれば，試験実施計画書の逸脱にはならないものとする。

得られた検体を遮光下で約 10 分間冷却遠心分離する（条件：4℃，約 1700×*g*）。血漿は検体採取後 1 時間以内にラベルを適切に貼付したスクリューキャップ付きポリプロピレンチューブへ移し，約-20℃で凍結保存する。

薬物濃度の測定は，UV-HPLC 法で測定する。

7.2　その他の臨床検査

以下に示す臨床検査を，投与前と，薬物動態用採血のための最終来院日に実施する。

血液学的検査	血液生化学的検査	尿検査
白血球数<br>白血球分画<br>（好中球，好酸球，好塩基球，リンパ球，単球）<br>ヘモグロビン<br>ヘマトクリット<br>赤血球数<br>血小板数	AST<br>ALT<br>アルカリフォスファターゼ<br>γGTP<br>LDH<br>総ビリルビン<br>アルブミン<br>クレアチニン<br>尿酸<br>カルシウム<br>ナトリウム<br>カリウム	pH<br>蛋白（定性）<br>糖（定性）<br>潜血（定性）

8 有害事象の報告

8.1 有害事象

試験中に観察されたまたは被験者から報告されたすべての有害事象は，試験薬との因果関係の有無にかかわらず報告する。

試験責任医師はすべての有害事象について，試験薬との因果関係，有害事象の転帰や重症度を判定するために必要な情報を収集する。

8.2 報告期間

試験薬の最終投与の 28 日後までとする。ただし，試験薬との因果関係が否定できない重篤な有害事象は，報告期間終了後であっても速やかに報告する。

8.3 検査値異常

有害事象として報告すべき検査値異常の判定基準は以下のとおりとする。

- 症状を伴う場合
- 追加検査が必要な場合，または内科的・外科的治療が必要な場合
- 試験薬の投与量変更または試験の中止の場合，もしくは臨床的に意味のある併用薬またはその他の治療を追加した場合
- 試験責任医師が有害事象と判断した場合

8.4 重篤な有害事象

重篤な有害事象または重篤な副作用とは，投与量を問わず発現した，以下に該当するあらゆる好ましくない医療上のできごとである。試験責任医師は個々の有害事象が重篤の基準に合致するかどうかを判断する。重篤な有害事象が発現した場合，実施国の規制および国際的規制に従い，適宜，緊急報告を行う。

- 死に至るもの
- 生命を脅かすもの
- 入院または入院期間の延長が必要となるもの
- 永続的または顕著な障害・機能不全に陥るもの

8.5 重症度評価

症例報告書の有害事象欄に記録する必要がある場合，試験責任医師は軽症（軽度），中等症（中等度），または重症（重度）を用いて有害事象の最高重症度を記録する。整合性をとるため，これらの強度のグレードを以下のように定義する。

軽症（軽度）	被験者の通常の機能を妨げない
中等症（中等度）	被験者の通常の機能をある程度妨げる
重症（重度）	被験者の通常の機能を顕著に妨げる

9. 統計解析

この試験で得られた日本人のリファブチンの血漿中濃度推移を評価する。

9.1 症例数の設定

【症例数の設定根拠】

過去の当院における患者数をもとに、目標例数は、日本人 HIV 感染者におけるリファブチンの薬物動態の検討において、十分に記述統計解析が実施できる例数とした。すなわち、1996 年より 2006 年に当院でリファブチンを使用した患者数は約 60 名で、年平均 10 名である。一般に、抗酸菌の初期治療時は抗 HIV 薬を併用しないことから、第 1 群の症例が抗 HIV 療法を開始した場合に引き続き第 2 群に参加すると仮定した場合、各群 10 名の計 20 名の参加を期待しうる。上記の評価方法および試験の実施可能性を考慮し，各群 10 例とした。

9.2 薬物動態解析

血漿中リファブチン濃度および薬物動態パラメータについて表，リスト，またはグラフで示し，記述的に要約する。

9.2.1 血漿中濃度

採血時間ごとに算術平均，標準偏差および変動係数（CV%）を算出する。この際，定量限界値未満の濃度については0（ゼロ）を代入する。

9.2.2 薬物動態パラメータの算出

１日目，１週目および１ヶ月目の血漿中リファブチン濃度の測定値から薬物動態パラメータを算出する。

最高血漿中濃度（C_{max}）および最高血漿中濃度到達時間（T_{max}）は，得られたデータの実測値を用いる。

消失速度定数（k_{el}）は，算出可能な場合は，消失相の血漿中濃度の対数を用いて最小二乗法により求め，血漿中濃度半減期（$t_{1/2}$）は，以下に示す式①により算出する。

$$t_{1/2} = \ln 2 / k_{el} \qquad ①$$

投与間隔（τ＝24 あるいは 48 時間）における血漿中濃度－時間曲線下面積（AUC_{τ}）は，投与後 τ 時間までの実測値から線形台形法を用いて算出する。

反復投与による累積係数（R_{ac}）は，1 週間目および１ヶ月目のほぼ定常状態における C_{max}（$C_{max,ss}$）または AUC_{τ}（$AUC_{\tau,ss}$）から，以下に示す式②-1，②-2 により算出する。

$$R_{ac} = C_{max,ss} / C_{max,（1日目）} \qquad ②\text{-}1$$

$$R_{ac} = AUC_{\tau,ss} / AUC_{\tau,（1日目）} \qquad ②\text{-}2$$

統計解析

血漿中濃度については採血時間毎に算術平均，標準偏差および変動係数(CV)を算出する。この際，定量限界値未満の濃度については 0（ゼロ）を代入し，過半数の被験者の血漿中濃度が定量限界値未満となった場合には，平均，標準偏差および CV は算出しない。

C_{max}， AUC_{τ}，R_{ac} については算術および幾何平均，標準偏差および CV を，T_{max} および $t_{1/2}$ については，算術，標準偏差および CV を算出する。

10. 品質管理および品質保証

試験の実施中，試験責任（分担）医師は定期的にモニタリングし，試験実施計画書および GCP が遵守されていることを確認する。試験責任医師および実施医療機関は，規制当局が照合のために原資料を直接閲覧できるようにする。

11. データの取扱いおよび記録の保存

11.1 症例報告書・電子的データ

本試験実施計画書における症例報告書（CRF）とは試験中のデータの収集方法に対応した紙の様式，電子的なデータの記録または両方とする。

試験に組み入れられた各被験者について症例報告書を作成する。作成済みの症例報告書の原本は，試験責任医師にすべての所有権があり，試験責任医師が認めた代理人または規制当局を除き，試験責任医師の文書による許可がない場合は，どのような形式でも第三者に開示してはならない。試験責任医師は常に症例報告書に記録された臨床データおよび臨床検査値の正確性および信頼性に対して最終責任を有する。

原資料とは試験実施医療機関に保存されている被験者に関する医療記録である。症例報告書に記録する情報はこれらの診療録と一致していなければならない。

12. 倫理

12.1 試験倫理委員会

実施にあたっては，試験実施計画書，試験実施計画書の改訂，同意文書，および広告等その他の関連文書について試験倫理委員会から事前に承認を取得することとする。ただし，被験者の緊急の危険を回避するために必要な変更が生じた場合，試験責任医師は，倫理委員会の承認を得る前に試験実施計画書の変更を行うことができる。

12.2 被験者への情報および同意

すべての試験関係者は被験者の個人情報を保護し，試験依頼者の書式，報告書，結果の公表，その他いかなる開示にも被験者名を含めない。データ移行の際には被験者の個人情報に対して高水準の守秘義務と保護を堅持する。

試験責任（分担）医師および試験協力者は，試験の内容および目的，参加に伴う可能性のある危険性について各被験者またはその代諾者に十分に説明しなければならない。試験責任（分担）医師は，試験にかかわる業務を実施する前に各被験者またはその代諾者から文書による同意を取得する。本試験で使用する同意説明文書および試験中に行う同意説明文書の変更は事前に倫理委員会の承認を得なければならない。各被験者が署名した同意文書の原本は医療機関にて保管する。

13. 試験依頼者が試験を中止する際の基準

倫理委員会の見解の変化，試験薬の安全性の問題，または試験責任（分担）医師の判断により，本試験を途中で中止する場合がある。

試験を途中で中止あるいは中断する場合，試験責任医師は可及的速やかに参加中のすべての被験者に連絡しなければならない。

14. 結果の公表

本試験から得られた結果は，国内外医薬雑誌または国内外の学術会議において，公表する。

15. 研究事務局

〒162-8655　東京都新宿区戸山 1-21-1
国立国際医療センター　エイズ治療・研究開発センター　田沼順子
TEL: 03-3202-7181　FAX: 03-3207-7198

16. 参考資料

i) ハリソン内科学，第 2 版（Harrison's Principles of Internal Medicine 16th edition）

ii) Mycobutin 米国添付文書

iii) American Thoracic Society. Diagnostic standards and classification of tuberculosis in adults and children. Am J Respir Crit Care Med (2000); 161: 1376 – 1395.

iv) American Thoracic Society / Centers for Disease Control and Prevention / Infectious Diseases Society of America. Treatment of tuberculosis. Am J Respir Crit Care Med (2003); 167: 603 – 662.

v) American Thoracic Society: Treatment of tuberculosis and tuberculosis infection in adults and children. Am J Respir Crit Care Med (1994); 149: 1359 – 1374.

vi) American Thoracic Society: Diagnosis and treatment of disease caused by nontuberculous mycobacteria. Am J Repir Crit Care Med (1997); 156: s1 – s25.

7) Boulanger et al. PK analysis of rifabutin given with lopinavir/ritonavir to persons co-infected with TB and HIV. Abstract 4th IAS 2007 conference. (2007) 22-25 July, Sydney, Australia. (http://www.ias2007.org/pag/Abstracts.aspx?SID=155&AID=3834)

8) Weiner et al. Association between Acquired Rifamycin Resistance and the Pharmacokinetics of Rifabutin and Isoniazid among Patients with HIV and Tuberculosis. Clin Infect Dis (2005); 40:1481–1491.
